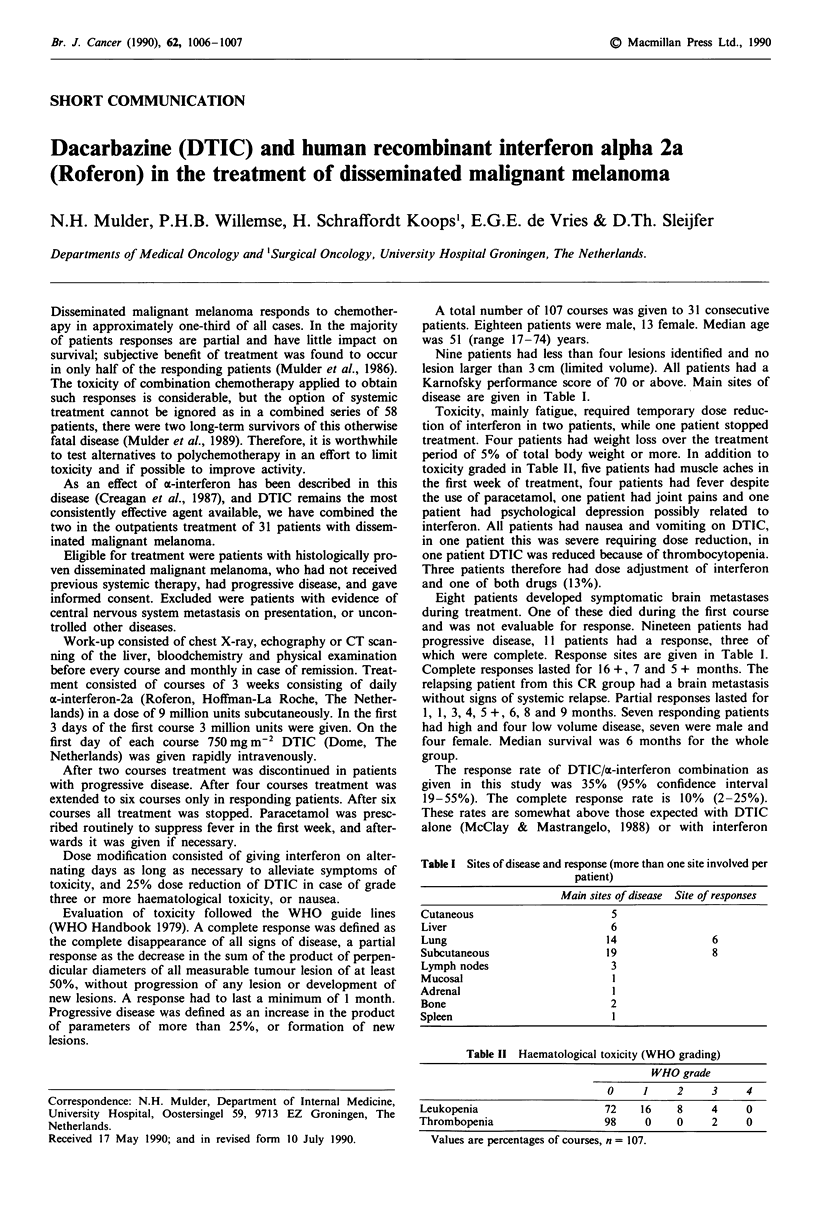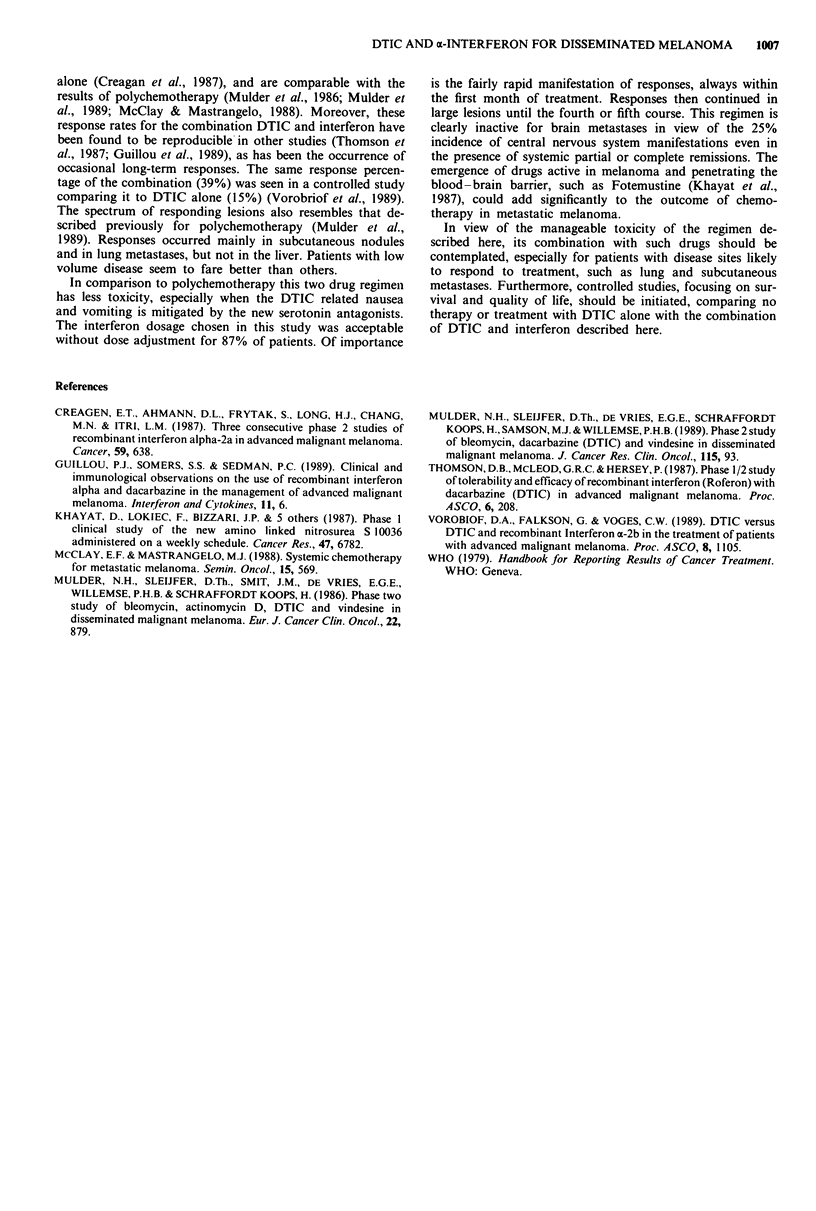# Dacarbazine (DTIC) and human recombinant interferon alpha 2a (Roferon) in the treatment of disseminated malignant melanoma.

**DOI:** 10.1038/bjc.1990.427

**Published:** 1990-12

**Authors:** N. H. Mulder, P. H. Willemse, H. Schraffordt Koops, E. G. de Vries, D. T. Sleijfer

**Affiliations:** Department of Medical Oncology, University Hospital Groningen, The Netherlands.


*Br. J. Cancer* (1990), **62**, 1006–1007 © Macmillan Press Ltd., 1990

SHORT COMMUNICATION

### Dacarbazine (DTIC) and human recombinant interferon alpha 2a (Roferon) in the treatment of disseminated malignant melanoma

N.H. Mulder, P.H.B. Willemse, H. Schraffordt Koops[1], E.G.E. de Vries & D.Th. Sleijfer

*Departments of Medical Oncology and [1]Surgical Oncology, University Hospital Groningen, The Netherlands.*

Disseminated malignant melanoma responds to chemotherapy in approximately one-third of all cases. In the majority of patients responses are partial and have little impact on survival; subjective benefit of treatment was found to occur in only half of the responding patients (Mulder *et al.*, 1986). The toxicity of combination chemotherapy applied to obtain such responses is considerable, but the option of systemic treatment cannot be ignored as in a combined series of 58 patients, there were two long-term survivors of this otherwise fatal disease (Mulder *et al.*, 1989). Therefore, it is worthwhile to test alternatives to polychemotherapy in an effort to limit toxicity and if possible to improve activity.

As an effect of α-interferon has been described in this disease (Creagan *et al.*, 1987), and DTIC remains the most consistently effective agent available, we have combined the two in the outpatients treatment of 31 patients with disseminated malignant melanoma.

Eligible for treatment were patients with histologically proven disseminated malignant melanoma, who had not received previous systemic therapy, had progressive disease, and gave informed consent. Excluded were patients with evidence of central nervous system metastasis on presentation, or uncontrolled other diseases.

Work-up consisted of chest X-ray, echography or CT scanning of the liver, bloodchemistry and physical examination before every course and monthly in case of remission. Treatment consisted of courses of 3 weeks consisting of daily α-interferon-2a (Roferon, Hoffman-La Roche, The Netherlands) in a dose of 9 million units subcutaneously. In the first 3 days of the first course 3 million units were given. On the first day of each course 750 mg m$^{-2}$ DTIC (Dome, The Netherlands) was given rapidly intravenously.

After two courses treatment was discontinued in patients with progressive disease. After four courses treatment was extended to six courses only in responding patients. After six courses all treatment was stopped. Paracetamol was prescribed routinely to suppress fever in the first week, and afterwards it was given if necessary.

Dose modification consisted of giving interferon on alternating days as long as necessary to alleviate symptoms of toxicity, and 25% dose reduction of DTIC in case of grade three or more haematological toxicity, or nausea.

Evaluation of toxicity followed the WHO guide lines (WHO Handbook 1979). A complete response was defined as the complete disappearance of all signs of disease, a partial response as the decrease in the sum of the product of perpendicular diameters of all measurable tumour lesion of at least 50%, without progression of any lesion or development of new lesions. A response had to last a minimum of 1 month. Progressive disease was defined as an increase in the product of parameters of more than 25%, or formation of new lesions.

Correspondence: N.H. Mulder, Department of Internal Medicine, University Hospital, Oostersingel 59, 9713 EZ Groningen, The Netherlands.
Received 17 May 1990; and in revised form 10 July 1990.

A total number of 107 courses was given to 31 consecutive patients. Eighteen patients were male, 13 female. Median age was 51 (range 17–74) years.

Nine patients had less than four lesions identified and no lesion larger than 3 cm (limited volume). All patients had a Karnofsky performance score of 70 or above. Main sites of disease are given in Table I.

Toxicity, mainly fatigue, required temporary dose reduction of interferon in two patients, while one patient stopped treatment. Four patients had weight loss over the treatment period of 5% of total body weight or more. In addition to toxicity graded in Table II, five patients had muscle aches in the first week of treatment, four patients had fever despite the use of paracetamol, one patient had joint pains and one patient had psychological depression possibly related to interferon. All patients had nausea and vomiting on DTIC, in one patient this was severe requiring dose reduction, in one patient DTIC was reduced because of thrombocytopenia. Three patients therefore had dose adjustment of interferon and one of both drugs (13%).

Eight patients developed symptomatic brain metastases during treatment. One of these died during the first course and was not evaluable for response. Nineteen patients had progressive disease, 11 patients had a response, three of which were complete. Response sites are given in Table I. Complete responses lasted for 16 +, 7 and 5 + months. The relapsing patient from this CR group had a brain metastasis without signs of systemic relapse. Partial responses lasted for 1, 1, 3, 4, 5 +, 6, 8 and 9 months. Seven responding patients had high and four low volume disease, seven were male and four female. Median survival was 6 months for the whole group.

The response rate of DTIC/α-interferon combination as given in this study was 35% (95% confidence interval 19–55%). The complete response rate is 10% (2–25%). These rates are somewhat above those expected with DTIC alone (McClay & Mastrangelo, 1988) or with interferon

**Table I** Sites of disease and response (more than one site involved per patient)

| | *Main sites of disease* | *Site of responses* |
|---|---|---|
| Cutaneous | 5 | |
| Liver | 6 | |
| Lung | 14 | 6 |
| Subcutaneous | 19 | 8 |
| Lymph nodes | 3 | |
| Mucosal | 1 | |
| Adrenal | 1 | |
| Bone | 2 | |
| Spleen | 1 | |

**Table II** Haematological toxicity (WHO grading)

| | *WHO grade* | | | | |
|---|---|---|---|---|---|
| | *0* | *1* | *2* | *3* | *4* |
| Leukopenia | 72 | 16 | 8 | 4 | 0 |
| Thrombopenia | 98 | 0 | 0 | 2 | 0 |

Values are percentages of courses, $n = 107$.

alone (Creagan *et al.*, 1987), and are comparable with the results of polychemotherapy (Mulder *et al.*, 1986; Mulder *et al.*, 1989; McClay & Mastrangelo, 1988). Moreover, these response rates for the combination DTIC and interferon have been found to be reproducible in other studies (Thomson *et al.*, 1987; Guillou *et al.*, 1989), as has been the occurrence of occasional long-term responses. The same response percentage of the combination (39%) was seen in a controlled study comparing it to DTIC alone (15%) (Vorobrief *et al.*, 1989). The spectrum of responding lesions also resembles that described previously for polychemotherapy (Mulder *et al.*, 1989). Responses occurred mainly in subcutaneous nodules and in lung metastases, but not in the liver. Patients with low volume disease seem to fare better than others.

In comparison to polychemotherapy this two drug regimen has less toxicity, especially when the DTIC related nausea and vomiting is mitigated by the new serotonin antagonists. The interferon dosage chosen in this study was acceptable without dose adjustment for 87% of patients. Of importance is the fairly rapid manifestation of responses, always within the first month of treatment. Responses then continued in large lesions until the fourth or fifth course. This regimen is clearly inactive for brain metastases in view of the 25% incidence of central nervous system manifestations even in the presence of systemic partial or complete remissions. The emergence of drugs active in melanoma and penetrating the blood–brain barrier, such as Fotemustine (Khayat *et al.*, 1987), could add significantly to the outcome of chemotherapy in metastatic melanoma.

In view of the manageable toxicity of the regimen described here, its combination with such drugs should be contemplated, especially for patients with disease sites likely to respond to treatment, such as lung and subcutaneous metastases. Furthermore, controlled studies, focusing on survival and quality of life, should be initiated, comparing no therapy or treatment with DTIC alone with the combination of DTIC and interferon described here.

### References

CREAGEN, E.T., AHMANN, D.L., FRYTAK, S., LONG, H.J., CHANG, M.N. & ITRI, L.M. (1987). Three consecutive phase 2 studies of recombinant interferon alpha-2a in advanced malignant melanoma. *Cancer*, **59**, 638.

GUILLOU, P.J., SOMERS, S.S. & SEDMAN, P.C. (1989). Clinical and immunological observations on the use of recombinant interferon alpha and dacarbazine in the management of advanced malignant melanoma. *Interferon and Cytokines*, **11**, 6.

KHAYAT, D., LOKIEC, F., BIZZARI, J.P. & 5 others (1987). Phase 1 clinical study of the new amino linked nitrosurea S 10036 administered on a weekly schedule. *Cancer Res.*, **47**, 6782.

McCLAY, E.F. & MASTRANGELO, M.J. (1988). Systemic chemotherapy for metastatic melanoma. *Semin. Oncol.*, **15**, 569.

MULDER, N.H., SLEIJFER, D.Th., SMIT, J.M., DE VRIES, E.G.E., WILLEMSE, P.H.B. & SCHRAFFORDT KOOPS, H. (1986). Phase two study of bleomycin, actinomycin D, DTIC and vindesine in disseminated malignant melanoma. *Eur. J. Cancer Clin. Oncol.*, **22**, 879.

MULDER, N.H., SLEIJFER, D.Th., DE VRIES, E.G.E., SCHRAFFORDT KOOPS, H., SAMSON, M.J. & WILLEMSE, P.H.B. (1989). Phase 2 study of bleomycin, dacarbazine (DTIC) and vindesine in disseminated malignant melanoma. *J. Cancer Res. Clin. Oncol.*, **115**, 93.

THOMSON, D.B., McLEOD, G.R.C. & HERSEY, P. (1987). Phase 1/2 study of tolerability and efficacy of recombinant interferon (Roferon) with dacarbazine (DTIC) in advanced malignant melanoma. *Proc. ASCO*, **6**, 208.

VOROBIOF, D.A., FALKSON, G. & VOGES, C.W. (1989). DTIC versus DTIC and recombinant Interferon α-2b in the treatment of patients with advanced malignant melanoma. *Proc. ASCO*, **8**, 1105.

WHO (1979). *Handbook for Reporting Results of Cancer Treatment.* WHO: Geneva.